# The Use of Sensitive Chemical Antibodies for Diagnosis: Detection of Low Levels of Epcam in Breast Cancer

**DOI:** 10.1371/journal.pone.0057613

**Published:** 2013-02-27

**Authors:** Sarah Shigdar, Christine Qian, Li Lv, Chunwen Pu, Yong Li, Lianhong Li, Manju Marappan, Jia Lin, Lifen Wang, Wei Duan

**Affiliations:** 1 School of Medicine, Deakin University, Waurn Ponds, Victoria, Australia; 2 Second Affiliated Hospital of Dalian Medical University, Dalian, China; 3 Dalian Sixth People’s Hospital, Dalian, China; 4 Cancer Care Centre, St. George Hospital, and St George Clinical School, Faculty of Medicine, University of New South Wales, Kensington, Australia; 5 Department of Pathology and Forensic Science, Dalian Medical University, Dalian, China; 6 Department of Histopathology, St John of God Pathology, Geelong, Victoria, Australia; Enzo Life Sciences, Inc., United States of America

## Abstract

EpCAM is expressed at low levels in a variety of normal human epithelial tissues, but is overexpressed in 70–90% of carcinomas. From a clinico-pathological point of view, this has both prognostic and therapeutic significance. EpCAM was first suggested as a therapeutic target for the treatment of epithelial cancers in the 1990s. However, following several immunotherapy trials, the results have been mixed. It has been suggested that this is due, at least in part, to an unknown level of EpCAM expression in the tumors being targeted. Thus, selection of patients who would benefit from EpCAM immunotherapy by determining EpCAM status in the tumor biopsies is currently undergoing vigorous evaluation. However, current EpCAM antibodies are not robust enough to be able to detect EpCAM expression in all pathological tissues. Here we report a newly developed EpCAM RNA aptamer, also known as a chemical antibody, which is not only specific but also more sensitive than current antibodies for the detection of EpCAM in formalin-fixed paraffin-embedded primary breast cancers. This new aptamer, together with our previously described aptamer, showed no non-specific staining or cross-reactivity with tissues that do not express EpCAM. They were able to reliably detect target proteins in breast cancer xenograft where an anti-EpCAM antibody (323/A3) showed limited or no reactivity. Our results demonstrated a more robust detection of EpCAM using RNA aptamers over antibodies in clinical samples with chromogenic staining. This shows the potential of aptamers in the future of histopathological diagnosis and as a tool to guide targeted immunotherapy.

## Introduction

Surgical pathology has become the ‘gold standard’ for the diagnosis of tumors [1]. When morphological features of the tumor are not adequate for a definitive diagnosis, immunohistochemistry (IHC) may be of benefit [2]. Indeed, antibodies have become an integral part of the pathology laboratory in the last 40 years [3]. Biomarkers are identified through the use of antibodies and molecular techniques, and help to identify particular characteristics of each tumor including those related to prognosis [4]. In addition to being an integral part of the diagnostic arsenal, IHC is also now being used to identify or differentiate those patients who are likely to benefit from certain directed or targeted therapies [5], [6]. Such is the case in HER2 positive breast cancer patients who benefit from treatment with trastuzumab [7].

EpCAM is a type I glycosylated membrane protein expressed at low levels in a variety of human epithelial tissues, but overexpressed in most solid cancers [8]. Indeed, its expression has been shown to be inversely related to the prognosis of cancer patients [8]. The detection of EpCAM on the surface of cancer cells is becoming increasingly important with the advent of anti-EpCAM immunotherapy. However, studies have shown that there is heterogeneity in the reactivity of antibodies against EpCAM, which could be due to different conformational states when the epitopes are differentially glycosylated [9], [10]. Antibody production is a time-consuming process that still relies heavily on the use of animals for their production, and while monoclonal antibodies are generally more pure than polyclonal antibodies, they may be contaminated by antibodies other than the one of interest when the ascites fluid is collected from the host animal [11]. An additional problem is that there may be batch-to-batch variation, with antibodies from different batches directed against the same epitope showing discrepancies in their staining [11]. Therefore, an alternative to antibodies that does not rely on *in vivo* production, and thus eliminates these variables would be highly advantageous. The generation of chemical antibodies, also known as aptamers, as an alternative to conventional antibodies is an area of research that shows promise in expanding the diagnostic and clinical arsenal in cancer diagnosis and treatment.

We recently reported the generation of a first aptamer against EpCAM that showed high sensitivity and specificity without any cross-reactivity [12]. Here we describe the capability of this aptamer, as well as a second aptamer against EpCAM, as an alternative to current EpCAM antibodies for chromogenic staining of formalin-fixed paraffin-embedded tumor tissues. Our results demonstrated that our aptamer was more sensitive than the EpCAM antibody in more than 90% of cases tested. These results have important clinical significance for breast cancer diagnosis and developing targeted therapies.

## Methods

### Ethics Statement

All animal procedures were approved by Deakin University Animal Welfare Committee and in accordance with the Australian Code of Practice for the Care and Use of Animals for Scientific Purposes.

### EpCAM Aptamers, Antibodies and Tissue Sections

Aptamers directed against EpCAM were generated as previously described [12]. Aptamers were chemically synthesized with 2′-fluoropyrimidine or 2-*O*-methyl-pyrimidine bases and a 5′-DY647 or TYE665 or FITC fluorescent tag and a 3′-inverted deoxthymidine (Dharmacon, Victoria, Australia): DT3∶5′-DY647- GCGACUGGUUACCCGGUCG-dT- 3′; Ep23∶5′-TYE665–ACGUAUCCCUUUUCGCGUA-dT-3′ or 5′-FITC–ACGUAUCCCUUUUCGC GUA-dT-3′; Control Aptamer: 5′-DY647-mGCmGACUmGmGUUmACCCmGmGUcmG-dT-3′. The monoclonal mouse anti-human EpCAM antibody (323/A3) was purchased from Abcam (Cambridge, MA). The secondary antibodies (goat anti-mouse- Alexa Fluor**®** 647, goat anti-fluorescein-horse radish peroxidase (HRP), or goat anti-mouse-HRP) were purchased from Life Technologies (Victoria, Australia), Abcam and Thermo Fisher Scientific (Victoria, Australia), respectively, while the 3,3′-diaminobenzidine (DAB) peroxidase substrate solution for chromogenic staining was purchased from Dako (K3467, Victoria, Australia). All antibodies and aptamers were optimized on positive and negative control tissues prior to the commencement of the study.

The formalin fixed paraffin embedded (FFPE) sections were generated from human breast cancer cell lines (T47D, MCF-7, MDA-MB-231), colon cancer cell line (HT-29) and glioblastoma cell line (U118-MG) implanted in BALB/c-Foxn1^nu^ mice for xenograft. HT-29 and U118-MG xenograft sections were used as positive and negative controls, respectively, throughout all staining procedures. The FFPE 4 µm tissue sections from breast cancer patient specimens were randomly selected from archived cases at Second Affiliated Hospital of Dalian Medical University, Dalian, China. These samples included eight cases of breast cancer (invasive ductal carcinoma (n = 7) and mucinous carcinoma (n = 1)) with matched lymph node metastases (Table 1). Negative controls were taken from normal liver tissue and xenografts derived from cell lines that do not express EpCAM were also used. Patients providing a tumor sample gave written informed consent to allow release of their archival tumor samples for research purposes. This study was also approved by institutional Human Research Ethics Committees.

**Table 1:** Clinical Details of Breast Tumor Cases.

Patient	Size of thetumor (cm)	Pathologicaldiagnosis	Numbers ofnodes involved	Clinico-pathologicstage	T	N	M
1	8	invasive ductal carcinoma	1	3a	3	1	0
2	4	invasive ductal carcinoma	8	3a	2	2	0
3	5	invasive ductal carcinoma	7	3c	4	3	0
4	6.5	mucinous carcinoma	2	3a	3	1	0
5	2	invasive ductal carcinoma	23	3c	1	3	0
6	10	invasive ductal carcinoma	43	3c	3	3	0
7	3	invasive ductal carcinoma	0	4	3	0	1
8	1	invasive ductal carcinoma	17	3c	1	3	0

Note: ‘T’: Tumor; N: Node; M: Metastasis.

### Quantitation of EpCAM Expression

EpCAM expression in the cell lines used in this study were analysed qualitatively by flow cytometry and quantitatively by Western analysis. The EpCAM-FITC antibody (Becton Dickerson, Victoria) was used for flow cytometric analysis of EpCAM expression. Furthermore, the binding sensitivity of the DT3 and Ep23 aptamers was confirmed against the T47D, MCF-7, MDA-MB-231 and HT-29 cell lines. Aptamers and antibody were incubated with the cells at 100 nM or 1∶10 dilution for 30 min at 37°C, washed three times and quantified using the flow cytometer. The expression of EpCAM protein in HT-29, T47D, MCF-7, MDA-MB-231 and U118-MG cells was analysed by Western analysis as previously described [13]. Fifteen microlitres of each sample was loaded onto a 12% NuPAGE Bis-Tris mini gel (Invitrogen) along with a Precision Plus dual colour protein standard (BioRad). Following electrophoresis for 45 min at 200 V, the protein was transferred to a nitrocellulose membrane (Invitrogen) and blocked with 5% skimmed milk for 3 h at 25°C, before being incubated with either anti-β-actin (Sigma) diluted 1∶2000, or the anti-EpCAM antibody, 323/A3 (Abcam) diluted 1∶250 in 1% skimmed milk, overnight at 4°C. Chemiluminescence was detected using an ImageQuant™ LAS 4000 Biomolecular Imager (GE Healthcare).

### Immunofluorescent Staining of Animal Xenograft Tissues

Paraffin embedded sections were deparaffinised with Histoclear and rehydrated through graded ethanols. Heat induced antigen retrieval was performed in a microwave oven using Tris-EDTA buffer (10 mM Tris, 1 mM EDTA, 0.05% Tween 20, pH 9.0) for 20 min and the slides were allowed to cool prior to blocking with 0.1 mg/mL tRNA, and 1 mg/mL bovine serum albumin (BSA) or 10% goat serum in phosphate buffered saline (PBS) for 1 h. Following the blocking step, slides were washed in PBS containing 0.1% Tween 20™ twice for 2 min prior to incubation with either EpCAM aptamer or antibody. Aptamers (100 nM) were prepared as previously described [12] and slides were incubated with aptamers in a solution of PBS containing 5 mM MgCl_2_, 0.1 mg/mL tRNA, 1 mg/mL BSA, 10% dextran sulphate and 500 µg/mL heparin for 15 min at 37°C. The slides were washed in PBS containing 0.1% Tween 20™ three times for 5 min each prior to incubation with Bisbenzimide Hoechst 33342 (Sigma, New South Wales, Australia) (3 µg/mL) for 10 min. Slides were then mounted using VECTASHIELD^®^ (Vector Laboratories, Burlingame, CA) and coverslipped.

For EpCAM antibody staining, tissue section preparation, antigen retrieval and blocking of non-specific binding sites were carried out under the same conditions as described above. The sections were probed with the EpCAM antibody 323/A3 (AB8601, Abcam, 1∶50 dilution) overnight at 4°C. After two washes with PBS-Tween-20™, the tissue sections were incubated with goat anti-mouse AlexaFluor 647 antibody (A21240, Life Technologies, 1∶200 dilution) for 2 h at room temperature then counterstained and coverslipped as described for the aptamers. Negative control slides were performed by replacing the primary antibody with PBS. Visualization was performed using a Fluoview FV10i laser scanning confocal microscope (Olympus). All images used for direct comparison were taken with identical exposure parameters and are presented without any digital contrast alteration or background subtraction.

### Chromogenic Staining of Human Breast Cancer and Normal Control Tissues

Normal breast, liver and breast cancer tissues were prepared as described above. Following antigen retrieval, endogenous peroxidase was blocked using 0.3% hydrogen peroxide for 20 minutes. Additional blocking with 10% goat serum and 0.1 mg/mL tRNA was carried out before aptamer (labeled with fluorescein) or antibody staining as previously described. A goat anti-mouse anti-fluorescein HRP secondary antibody (AB6656, Abcam, 1∶100 dilution) or a goat anti-mouse HRP secondary antibody (31430, Thermo Fisher Scientific, 1∶500 dilution) was used to develop the signal from the aptamer or 323/A3 antibody for 2 h, respectively. The slides were then treated with DAB peroxidase substrate solution (Dako) for color development at room temperature for 10 min and counterstained with haematoxylin solution for 5 min following routine laboratory protocol. Stained sections were examined under a light microscope equipped with an Olympus SC20 camera (Victoria, Australia). As a negative control, aptamers were omitted from the staining reaction. All staining experiments also included positive and negative control slides (HT-29 and U118-MG cell line xenografts, respectively). Antigen expression was defined as specific when a staining signal was present on the tumor cell membrane. EpCAM expression was evaluated and compared to the staining intensity of the control cell line (HT-29). Antigen staining was distinguished as ‘−’ for no staining; ‘1+’ for faint incomplete staining; ‘2+’ for moderate complete membrane staining; or ‘3+’ for strong complete membrane staining.

## Results

### Specific Staining of Cancer Cells in Cancer Xenografts by EpCAM Aptamers

**Figure 1:**
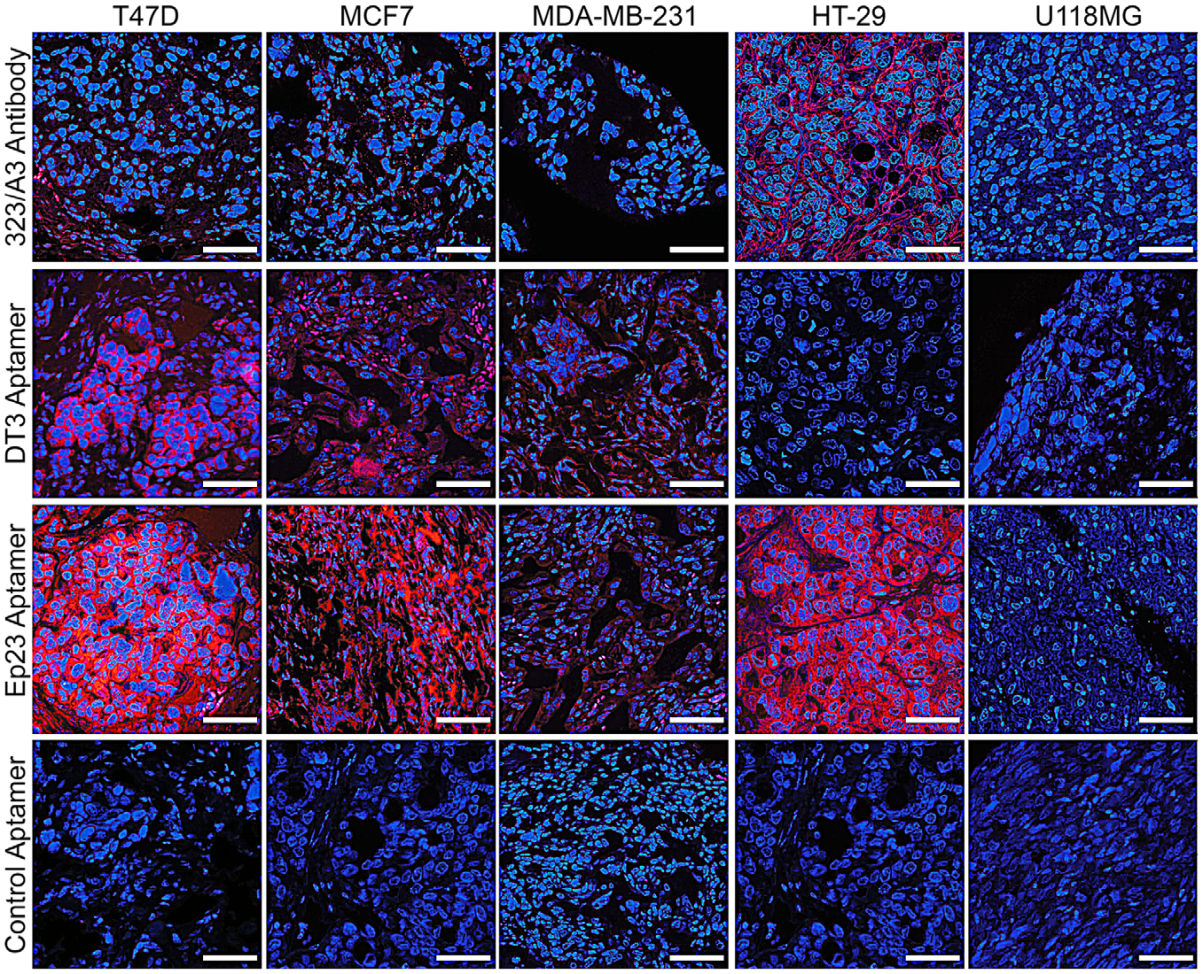
Detection of EpCAM in paraffin embedded tissue using aptamers and antibodies. Immunofluorescence staining of breast cancer (T47D, MCF7 and MDA-MB-231), colon cancer (HT-29) and glioblastoma (U118MG) xenograft tumors by EpCAM antibody, 323/A3, and EpCAM aptamers, DT3 and Ep23, and control aptamer (Blue: nuclei; Red: EpCAM positive staining). Aptamer staining was performed for 15 min at 37°C, while 323/A3 staining was performed at 4°C overnight. All fluorescent images were taken under a confocal microscope with×60 magnification. Images are representative of at least three separate experiments. Scale bar: 50 µm.

Protocol optimization was performed using breast and colon cancer xenograft tissues and fluorescently labeled aptamers or the EpCAM antibody 323/A3. When incubated in the presence of dextran sulphate and heparin, the anti-EpCAM aptamers DT3 and Ep23 showed highly sensitive staining for T47D, MCF7 and MDA-MB-231 human breast cancer xenografts at a concentration of 100 nM for 15 min at 37°C (Figure 1). Indeed, the staining pattern correlates well with the reported level of EpCAM expression on the surface of breast cancer cells [14], as demonstrated by weaker staining of the MDA-MB-231 xenograft sections that express much lower amounts of EpCAM than T47D and MCF7, and as confirmed by flow cytometric analysis and Western analysis (Figure S1). Also, staining was shown to be highly specific, with no staining identified for the U118MG xenograft (EpCAM-negative), or the control aptamer on any of the xenograft sections (Figure 1).

### Aptamers are More Sensitive at Detecting EpCAM than Antibodies in Cancer Xenografts

As shown in Figure 1 and Table 2, the intensity of antibody staining for the three different breast cancer xenografts using an anti-EpCAM antibody 323/A3 was much weaker than that of the aptamers, in particular aptamer Ep23. However, the antibody 323/A3 did show moderate to strong staining of the HT-29 colorectal cancer xenograft. These results indicate our aptamers are much more sensitive than one of the standard anti-EpCAM antibodies in use in pathology laboratories [10], [15], [16], [17]. Among all the human cancer cell lines studied here, MDA-MB-231 cells express the lowest amounts of EpCAM (1.7×10^3^ binding sites/cell in MDA-MB-231 vs 222.1×10^3^ binding sites/cell in MCF7 [14]. Interestingly, both of our aptamers were able to successfully detect EpCAM in FFPE sections of a xenograft tumor of MDA-MB-231, while the EpCAM antibody showed negligible staining (Figure 1), demonstrating the higher sensitivity of our aptamers.

**Table 2:** Quantification of anti-EpCAM staining in cell line xenografts using DT3, Ep23 and control aptamers and 323/A3 antibody by immunofluorescence.

	Cell Lines				
	T47D	MCF7	MDA-MB-231	HT-29	U118MG
323/A3 Antibody	1+	1+	−	2+/3+	−
DT3 Aptamer	2+	1+	1+	−	−
Ep23 Aptamer	3+	2+	1+	3+	−
Control Aptamer	−	−	−	−	−

‘−’ no staining; ‘+’ faint incomplete staining (negative); ‘++’ moderate complete membrane staining (equivocal); ‘+++’ strong complete membrane staining (positive) [45].

### Robust Detection of EpCAM in Clinical Samples

**Figure 2:**
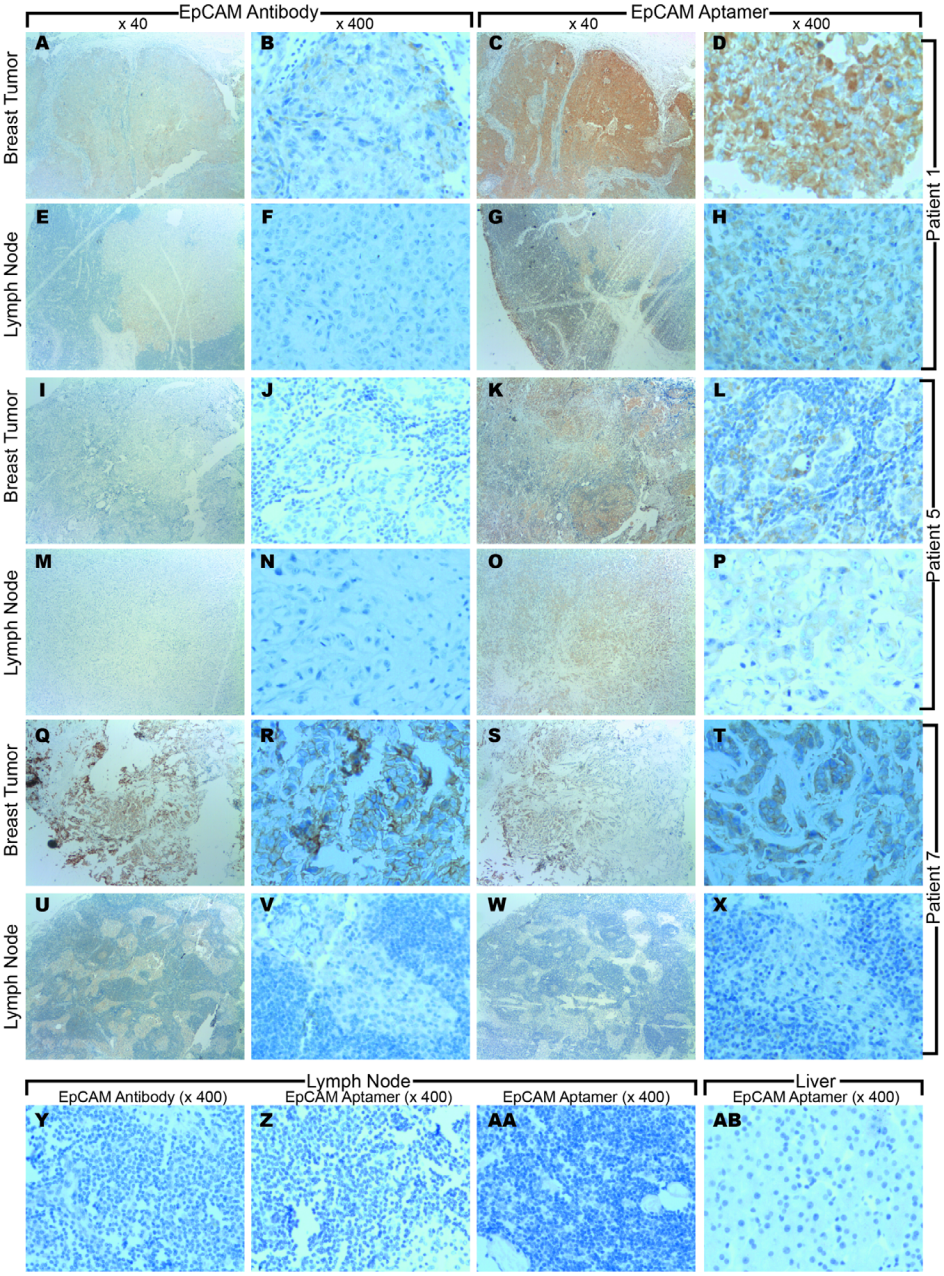
Tissue immunostaining of breast cancer and lymph node metastasis by EpCAM antibody and aptamer. A – H: EpCAM antibody immunostaining was weaker in both the breast tumor (A (× 40), B (× 400) and lymph node (E (× 40), F (× 400) in comparison to EpCAM aptamer immunostaining in patient 1 (Breast tumor C (×40), D (×400); and lymph node G (× 40), H (× 400); I – P No immunostaining was observed with the EpCAM antibody in the breast tumor (I (× 40), J (× 400) or the lymph node (M (× 40), N (× 400)) while the EpCAM aptamer showed a strong positive signal in both the breast (K (× 40), L (× 400)) and lymph node (O (× 40), P (× 400)) in patient 5; Q – X Immunostaining with EpCAM antibody was stronger in the breast tumor (Q (× 40), R (× 400)) but not the lymph node (U (× 40), V (× 400)) than the EpCAM aptamer (Breast tumor S (× 40), T (× 400); and lymph node W (× 40), X (× 400)) in patient 7; Y – AB Representative images of negative control tissues. Patient 4 showed negative areas of lymphocyte staining within the lymph node by EpCAM antibody (Y (× 400)) and EpCAM aptamer (Z (× 400)); Patient 2 showed normal regions of lymph node that was negative by EpCAM aptamer (AA (× 400)); Normal liver sample negative for EpCAM by EpCAM aptamer (AB (× 400)). All pictures were taken under a light microscope with × 40 magnification and × 400 magnification (taken from the center of the × 40 magnification).

Since one of our aptamers demonstrated superior staining using a fluorescence detection system, a chromogenic staining protocol using this aptamer-substrate system suitable for light microscopy evaluation was optimized. Archived FFPE histopathology samples from eight breast cancer patients with matched tumor and lymph node metastasis sections were independently evaluated for aptamer and antibody staining intensity. In 14 of the 16 sections, the aptamer staining intensity of tumor cells was superior to that seen with the antibody (Table 3, Figure 2). Indeed, in a number of breast tumors, no obvious staining was observed with the EpCAM antibody (Figure 2I, J), while the aptamer showed a moderate positive signal (Figure 2K, L). One lymph node could not be assessed as only 1% of the lymph node showed tumor and this area was missing from the section for evaluation. This case was used as a negative control (Figure 2Y, Z) showing that both the antibody and aptamer stained specific EpCAM positive cells, and were not prone to non-specific staining. Interestingly, only one breast ductal carcinoma showed a slightly superior staining with the antibody compared to the aptamer (Figure 2Q, R). The specificity of our EpCAM aptamer was demonstrated by no cross-reactive staining of the normal cells or lymphocytes within the tissue sections (Figure 2Z, AA). To confirm that non-specific binding was not the cause of the increased sensitivity seen with our aptamer, normal human liver (EpCAM negative) sections were also evaluated for staining and all showed no discernible DAB signals (Figure 2AB). Staining of both positive and negative control slides showed appropriate reactivity indicating the correct optimization of both antibody and aptamer staining protocols (Figure S2).

**Table 3:** Immunostaining Comparison of EpCAM Antibody and Aptamer.

	Breast Tumor		Lymph Node	
Case Number	Antibody Staining	Aptamer Staining	Antibody Staining	Aptamer Staining
**1**	2+	3+	1+/2+	2+
**2**	0	2+	0	1+/2+
**3**	0	2+/3+	1+	2+
**4**	0/1+	2+	No Tumor	1+/2+
**5**	0	2+/3+	0	1+/2+
**6**	0	2+/3+	1+	1+/2+
**7**	3+	2+	1+	1+/2+
**8**	0	2+	0	1+/2+

‘−’ no staining; ‘+’ faint incomplete staining (negative); ‘++’ moderate complete membrane staining (equivocal); ‘+++’ strong complete membrane staining (positive) [45].

## Discussion

A sensitive and reliable method for evaluating the expression of cancer biomarkers in tissue sections is essential for personalized medicine. EpCAM is a cancer biomarker with its overexpression documented in both primary and metastatic breast cancers [18]. Increasing EpCAM expression is associated with adverse clinical outcomes in these patients, especially in metastases [19], [20], [21]. While it has been suggested that the level of EpCAM expression be tested in cancer patients [19], [22], there is no consensus on the method to be used [19]. A sensitive and reliable method for evaluating EpCAM expression is essential for successful immunotherapy [22].

Aptamers are small single-stranded DNA or RNA molecules that are also known as chemical antibodies. This is because of their ability to fold into complex three-dimensional shapes and bind to their target in a similar manner to conventional antibodies [23], [24], [25], [26], [27]. One key benefit of these ligands over antibodies is that they are chemically synthesized, thus reducing many of the disadvantages of antibodies. Aptamers are produced via a process known as the systematic evolution of ligands by exponential enrichment (SELEX), which through iterative rounds, produces a molecule highly specific for its target [23], [24]. An additional benefit of aptamers is that they can be modified or functionalized without loss of affinity [28], [29]. However, while aptamers show great promise, there have been limited reports of their use as probes for IHC [30], [31], with only one report to date of the use of aptamers for histopathological diagnosis in paraffin embedded tissues [30]. However, aptamers can be generated to any protein target and substituted for antibodies in virtually any application [32], [33], [34]. It has been suggested that one of the difficulties associated with substituting aptamers for antibodies in histopathological protocols is the potential for non-specific staining due to electrostatic attraction of these polyanion nucleic acid aptamers to positively charged sites, such as histones, in nucleis [31]. This could explain the paucity of published data on the use of these chemical antibodies for histological diagnosis.

Here we demonstrate that aptamers are also suitable for histological diagnosis. While extensive optimization was required in the attempt to use these ligands as chemical antibodies, the realization that these probes are nucleic acids and therefore might require a combinational approach of both conventional antibody staining, and methods similar to those used for *in situ* hybridization, led to successful results [35], [36], [37]. The use of dextran sulphate has been shown to accelerate the rate of nucleic acid hybridization [35] thus reducing the time required for aptamer incubation, while heparin has been shown to reduce background binding in hybridization procedures [37]. When either of these two reagents was omitted from the hybridization buffer, positive staining was not achieved, even when incubation time was increased. Indeed, this approach was successful for all the aptamers used for EpCAM staining. We anticipate that this improved protocol will prove to be highly effective for staining other biomarkers in FFPE tissues using aptamers and we are currently investigating this hypothesis.

The sharp contrast between the ability of our EpCAM aptamers to detect low levels of EpCAM antigen in paraffin embedded tissues and the lack of staining by conventional antibodies of the same tumors could be attributed to a number of factors: 1) Aptamers are 10–20 times smaller (6 kDa vs 150 kDa) than antibodies and it is therefore possible that higher numbers of aptamers can bind to a given target molecule compared to antibodies; 2) It has been well documented that antigen density is a critical factor in the effectiveness of the detection of antigens by monoclonal antibodies [38]. Therefore, it is possible that low levels of EpCAM expression may be missed during diagnosis using conventional antibody staining. This phenomenon was reported for the estrogen receptor in an external quality control scheme (UK-NEQAS-ICC), where tumors expressing low levels of estrogen receptors were often falsely reported as negative [39]. The staining intensity and sensitivity of our aptamer would improve detection in these cases. EpCAM staining with our aptamer showed superior results when compared to the antibody in both human xenograft tumors via fluorescently labeled aptamer and in primary human breast cancers using chromogenic staining. These results are consistent with findings by others that aptamers display a high level of sensitivity and low background staining [12], [30]. A recent paper has shown that 80% of invasive ductal breast carcinomas are EpCAM positive [19]. Given the results from the present study, it is possible that the level of EpCAM positivity is underestimated in primary and metastatic tumors using conventional antibodies. It has been suggested that EpCAM expression in triple-negative breast cancer may be a potential target for immunotherapy, given its resistance to current targeted therapies, such as endocrine treatment and trastuzumab [40]. Therefore, accurate and quantitative assessment of the level of EpCAM expression can better inform the clinician when deciding on targeted treatment options.

To confirm the sensitivity of our aptamers, we initially chose several breast cancer cell lines that have a known level of EpCAM expression on their cell surface, as well as a colon cancer cell line that has a known high level of EpCAM expression. It has been reported that HT-29 has 2.3 million EpCAM binding sites on the cell surface [41], while T47D and MCF-7 have similar levels of expression (0.22 million binding sites per cell) [14], [42]. These results were confirmed by Western analysis (Figure S1 I & J). MDA-MB-231 has a very low level of EpCAM expression, with a reported 1.7 thousand EpCAM binding sites per cell [14]. The EpCAM antibody used in this study had an insufficient sensitivity level to detect EpCAM in the MDA-MB-231 xenograft model. However, when fully optimized, our aptamer staining protocol was able to stain this weakly positive EpCAM expressing xenograft tissue as well as HT-29 xenograft tissue with a dynamic range of 3 orders of magnitude, thus confirming the high sensitivity of our detection system in paraffin-embedded tissues.

While both aptamers showed a higher sensitivity with the breast cancer xenografts, aptamer DT3 did not show any positive staining of the HT-29 xenograft tissues. The binding affinity of DT3 for HT-29 is 158 nM [12], while Ep23 shows a 4-fold higher binding affinity for HT-29 (37 nM). This difference in binding affinities is most probably the cause of the lack of any noticeable staining due to weaker intermolecular bonds between the aptamer and the protein. This discrepancy in staining is not a phenomenon solely associated with aptamers and is observed with antibodies as well. In the case of EpCAM detection, the Ber-EP4 antibody has been shown to display false negative results in paraffin embedded cell blocks [43]. Therefore, as with antibodies, a negative result needs to be confirmed with additional aptamers to confirm the lack of expression, rather than the lack of a reaction [44].

Having confirmed the sensitivity of our aptamers on a range of human cancer cell lines expressing variable levels of EpCAM, we sought to determine the specificity of our aptamers. Initially, this was confirmed using a glioblastoma xenograft that does not express EpCAM [12]. In all cases there was no detectable signal from either of the aptamers. Once the chromogenic staining protocol was optimized, several controls testing specificity were used. Traditional negative controls, such as our glioblastoma xenograft and normal human liver, were incorporated into each staining experiment. An additional negative control, the omission of the aptamer from the staining reaction, was performed for all tumor cases to confirm the enhanced sensitivity of our aptamer was not due to non-specific binding of the chromogen. As well, staining of normal cells was assessed in all lymph node cases. Specificity was confirmed by the lack of staining of lymphocytes within each assessed sections with positive staining. The inclusion of positive and negative control cancer cell line xenografts provided additional evidence on the specificity of both the aptamer and antibody. The reliability and reproducibility was demonstrated by the consistent staining patterns observed with the positive and negative xenograft tissue sections throughout the entire study.

Antibodies have previously been considered to be sensitive for antigen detection. However, here we have shown that when both the staining conditions for antibodies and aptamers are fully optimized, aptamers could be more sensitive than conventional antibodies for the detection of EpCAM. As well, the incubation times were dramatically reduced (overnight for 323/A3 antibody vs 15 min for Ep23 aptamer), showing optimal staining with a shorter reaction time. Additional studies are required to validate this aptamer as a histological tool for the diagnosis of EpCAM expression. However, the results from this study demonstrate that aptamers are not only capable of functioning as chemical antibodies, but are also more sensitive than conventional antibodies, at least in the case of EpCAM. As aptamers can be chemically synthesized and modified easily, they suffer no batch-to-batch variation, and as they are much more stable than protein antibodies, this study paves the way for the application of these molecular probes in future histopathological diagnosis and potentially for therapeutic applications. Aptamers can provide a robust and cost-effective tool to translate discoveries from biomarker and cancer stem cell research into pathology diagnostic practice to better stratify patients for personalized medicine.

## Supporting Information

Figure S1 **Determination of EpCAM expression by flow cytometric analysis and Western analysis.** Flow cytometry was used to confirm EpCAM expression by EpCAM-FITC antibody and DT3 and Ep23 aptamers. A: Flow cytometry analysis of EpCAM expression of T47D with DT3 and Ep23 aptamers; B Flow cytometry analysis of EpCAM expression of T47D with EpCAM-FITC antibody; C: Flow cytometry analysis of EpCAM expression of MCF-7 with DT3 and Ep23 aptamers; D: Flow cytometry analysis of EpCAM expression of MCF-7 with EpCAM-FITC antibody; E: Flow cytometry analysis of EpCAM expression of MDA-MB-231 with DT3 and Ep23 aptamers; F Flow cytometry analysis of EpCAM expression of MDA-MB-231 with EpCAM-FITC antibody; G: Flow cytometry analysis of EpCAM expression of HT-29 with DT3 and Ep23 aptamers; H Flow cytometry analysis of EpCAM expression of HT-29 with EpCAM-FITC antibody. Black: negative control; Blue: DT3 aptamer (A, C, E, G); Red: Ep23 aptamer (A, C, E, G); Purple: EpCAM antibody (B, D, F, H). I: EpCAM expression was confirmed by Western analysis using the 323/A3 antibody; J: Relative expression of EpCAM was compared to β-actin.
(TIF)

Figure S2 **Representative images of positive and negative control slides for chromogenic staining.** HT-29 and U118MG tissue sections were stained with either EpCAM antibody or Ep23 aptamer as part of each staining experiment of clinical breast cancer cases to confirm specificity of each staining reaction.
(TIF)

## References

[1] Rodriguez-Canales J, Eberle FC, Jaffe ES, Emmert-Buck MR (2011) Why is it crucial to reintegrate pathology into cancer research? BioEssays 33: 490–498. 21590787 10.1002/bies.201100017 PMC6377259

[2] Idikio HA (2009) Immunohistochemistry in diagnostic surgical pathology: contributions of protein life-cycle, use of evidence-based methods and data normalization on interpretation of immunohistochemical stains. Int J Clin Exp Pathol 3: 169–176. 20126585 PMC2809997

[3] Stone MJ, Aronoff BE, Evans WP, Fay JW, Lieberman ZH, et al (2003) History of the Baylor Charles A. Sammons Cancer Center. Proceedings (Baylor University Medical Center) 16: 30–58. 16278720 10.1080/08998280.2003.11927886 PMC1200808

[4] Bhatt AN, Mathur R, Farooque A, Verma A, Dwarakanath BS (2010) Cancer biomarkers - current perspectives. Indian J Med Res 132: 129–149. 20716813

[5] Lindblom A, Liljegren A (2000) Regular review: tumour markers in malignancies. BMJ 320: 424–427. 10669448 10.1136/bmj.320.7232.424 PMC1117546

[6] Sharma S (2009) Tumor markers in clinical practice: General principles and guidelines. Indian J Med Paediatr Oncol 30: 1–8. 20668599 10.4103/0971-5851.56328 PMC2902207

[7] Arteaga CL, Sliwkowski MX, Osborne CK, Perez EA, Puglisi F, et al (2012) Treatment of HER2-positive breast cancer: current status and future perspectives. Nat Rev Clin Oncol 9: 16–32. 10.1038/nrclinonc.2011.177 22124364

[8] Patriarca C, Macchi RM, Marschner AK, Mellstedt H (2012) Epithelial cell adhesion molecule expression (CD326) in cancer: a short review. Cancer Treat Rev 38: 68–75. 21576002 10.1016/j.ctrv.2011.04.002

[9] Antolovic D, Galindo L, Carstens A, Rahbari N, Buchler MW, et al (2010) Heterogeneous detection of circulating tumor cells in patients with colorectal cancer by immunomagnetic enrichment using different EpCAM-specific antibodies. BMC Biotechnol 10: 35. 20426872 10.1186/1472-6750-10-35 PMC2868015

[10] Balzar M, Briaire-de Bruijn IH, Rees-Bakker HA, Prins FA, Helfrich W, et al (2001) Epidermal growth factor-like repeats mediate lateral and reciprocal interactions of Ep-CAM molecules in homophilic adhesions. Mol Cell Biol 21: 2570–2580. 11259604 10.1128/MCB.21.7.2570-2580.2001 PMC86888

[11] Bordeaux J, Welsh A, Agarwal S, Killiam E, Baquero M, et al (2010) Antibody validation. Biotechniques 48: 197–209. 20359301 10.2144/000113382 PMC3891910

[12] Shigdar S, Lin J, Yu Y, Pastuovic M, Wei M, et al (2011) RNA aptamer against a cancer stem cell marker epithelial cell adhesion molecule. Cancer Sci 102: 991–998. 21281402 10.1111/j.1349-7006.2011.01897.x

[13] Yeong SS, Zhu Y, Smith D, Verma C, Lim WG, et al (2006) The last 10 amino acid residues beyond the hydrophobic motif are critical for the catalytic competence and function of protein kinase Calpha. J Biol Chem 281: 30768–30781. 16895917 10.1074/jbc.M511278200

[14] Prang N, Preithner S, Brischwein K, Goster P, Woppel A, et al (2005) Cellular and complement-dependent cytotoxicity of Ep-CAM-specific monoclonal antibody MT201 against breast cancer cell lines. Br J Cancer 92: 342–349. 15655555 10.1038/sj.bjc.6602310 PMC2361858

[15] Winter MJ, Nagtegaal ID, van Krieken JH, Litvinov SV (2003) The epithelial cell adhesion molecule (Ep-CAM) as a morphoregulatory molecule is a tool in surgical pathology. Am J Pathol 163: 2139–2148. 14633587 10.1016/S0002-9440(10)63570-5 PMC1892395

[16] Songun I, Litvinov SV, van de Velde CJ, Pals ST, Hermans J, et al (2005) Loss of Ep-CAM (CO17–1A) expression predicts survival in patients with gastric cancer. Br J Cancer 92: 1767–1772. 15870832 10.1038/sj.bjc.6602519 PMC2362035

[17] Hough CD, Sherman-Baust CA, Pizer ES, Montz FJ, Im DD, et al (2000) Large-scale serial analysis of gene expression reveals genes differentially expressed in ovarian cancer. Cancer Res 60: 6281–6287. 11103784

[18] van der Gun BT, Melchers LJ, Ruiters MH, de Leij LF, McLaughlin PM, et al (2010) EpCAM in carcinogenesis: the good, the bad or the ugly. Carcinogenesis 31: 1913–1921. 20837599 10.1093/carcin/bgq187

[19] Spizzo G, Fong D, Wurm M, Ensinger C, Obrist P, et al (2011) EpCAM expression in primary tumour tissues and metastases: an immunohistochemical analysis. J Clin Pathol 64: 415–420. 21415054 10.1136/jcp.2011.090274 PMC3088404

[20] Cimino A, Halushka M, Illei P, Wu X, Sukumar S, et al (2010) Epithelial cell adhesion molecule (EpCAM) is overexpressed in breast cancer metastases. Breast Cancer Res Treat 123: 701–708. 20012351 10.1007/s10549-009-0671-z PMC3042397

[21] Sankpal NV, Mayfield JD, Willman MW, Fleming TP, Gillanders WE (2011) Activator protein 1 (AP-1) contributes to EpCAM-dependent breast cancer invasion. Breast Cancer Res 13: R124. 22132731 10.1186/bcr3070 PMC3326566

[22] Gires O, Bauerle PA (2010) EpCAM as a target in cancer therapy. J Clin Oncol 28: e239–240; author reply e241–232. 10.1200/JCO.2009.26.8540 20385979

[23] Ellington AD, Szostak JW (1990) In vitro selection of RNA molecules that bind specific ligands. Nature 346: 818–822. 1697402 10.1038/346818a0

[24] Tuerk C, Gold L (1990) Systematic evolution of ligands by exponential enrichment: RNA ligands to bacteriophage T4 DNA polymerase. Science 249: 505–510. 2200121 10.1126/science.2200121

[25] Shigdar S, Ward AC, De A, Yang CJ, Wei M, et al (2011) Clinical applications of aptamers and nucleic acid therapeutics in haematological malignancies. Br J Haematol 155: 3–13. 21810089 10.1111/j.1365-2141.2011.08807.x

[26] Rayburn ER, Zhang R (2008) Antisense, RNAi, and gene silencing strategies for therapy: mission possible or impossible? Drug Discov Today 13: 513–521. 18549978 10.1016/j.drudis.2008.03.014 PMC2497463

[27] Stoltenburg R, Reinemann C, Strehlitz B (2007) SELEX-A (r)evolutionary method to generate high-affinity nucleic acid ligands. Biomol Eng 24: 381–403. 17627883 10.1016/j.bioeng.2007.06.001

[28] Farokhzad OC, Jon S, Khademhosseini A, Tran TN, Lavan DA, et al (2004) Nanoparticle-aptamer bioconjugates: a new approach for targeting prostate cancer cells. Cancer Res 64: 7668–7672. 15520166 10.1158/0008-5472.CAN-04-2550

[29] Das M, Mohanty C, Sahoo SK (2009) Ligand-based targeted therapy for cancer tissue. Expert Opin Drug Deliv 6: 285–304. 19327045 10.1517/17425240902780166

[30] Zeng Z, Zhang P, Zhao N, Sheehan AM, Tung CH, et al (2010) Using oligonucleotide aptamer probes for immunostaining of formalin-fixed and paraffin-embedded tissues. Mod Pathol 23: 1553–1558. 20693984 10.1038/modpathol.2010.151 PMC3159180

[31] Gupta S, Thirstrup D, Jarvis TC, Schneider DJ, Wilcox SK, et al (2011) Rapid histochemistry using slow off-rate modified aptamers with anionic competition. Appl Immunohistochem Mol Morphol 19: 273–278. 21217521 10.1097/PAI.0b013e3182008c29

[32] Lassalle HP, Marchal S, Guillemin F, Reinhard A, Bezdetnaya L (2012) Aptamers as remarkable diagnostic and therapeutic agents in cancer treatment. Curr Drug Metab. 10.2174/138920012802850038 22380008

[33] Baird GS (2010) Where are all the aptamers? Am J Clin Pathol 134: 529–531. 20855632 10.1309/AJCPFU4CG2WGJJKS

[34] Keefe AD, Pai S, Ellington A (2010) Aptamers as therapeutics. Nat Rev Drug Discov 9: 537–550. 20592747 10.1038/nrd3141 PMC7097324

[35] Wahl GM, Stern M, Stark GR (1979) Efficient transfer of large DNA fragments from agarose gels to diazobenzyloxymethyl-paper and rapid hybridization by using dextran sulfate. Proc Natl Acad Sci U S A 76: 3683–3687. 291033 10.1073/pnas.76.8.3683 PMC383897

[36] van Gijlswijk RP, Wiegant J, Raap AK, Tanke HJ (1996) Improved localization of fluorescent tyramides for fluorescence in situ hybridization using dextran sulfate and polyvinyl alcohol. J Histochem Cytochem 44: 389–392. 8601698 10.1177/44.4.8601698

[37] Singh L, Jones KW (1984) The use of heparin as a simple cost-effective means of controlling background in nucleic acid hybridization procedures. Nucleic Acids Res 12: 5627–5638. 6087294 10.1093/nar/12.14.5627 PMC320019

[38] Kataria S, Middha A, Sandhu P, Kapoor B (2011) Monoclonal antibody as anti cancer agents. IJPBA 2: 1346–1356.

[39] Rhodes A, Jasani B, Balaton AJ, Miller KD (2000) Immunohistochemical demonstration of oestrogen and progesterone receptors: correlation of standards achieved on in house tumours with that achieved on external quality assessment material in over 150 laboratories from 26 countries. J Clin Pathol 53: 292–301. 10823126 10.1136/jcp.53.4.292 PMC1731173

[40] Schmidt M, Hasenclever D, Schaeffer M, Boehm D, Cotarelo C, et al (2008) Prognostic effect of epithelial cell adhesion molecule overexpression in untreated node-negative breast cancer. Clin Cancer Res 14: 5849–5855. 18794096 10.1158/1078-0432.CCR-08-0669

[41] Thurber GM, Weissleder R (2011) Quantitating antibody uptake in vivo: conditional dependence on antigen expression levels. Mol Imaging Biol 13: 623–632. 20809210 10.1007/s11307-010-0397-7 PMC3000888

[42] Heine M, Freund B, Nielsen P, Jung C, Reimer R, et al (2012) High interstitial fluid pressure is associated with low tumour penetration of diagnostic monoclonal antibodies applied for molecular imaging purposes. PLoS One 7: e36258. 22590529 10.1371/journal.pone.0036258 PMC3348149

[43] Arora R, Agarwal S, Mathur SR, Verma K, Iyer VK, et al (2011) Utility of a limited panel of calretinin and Ber-EP4 immunocytochemistry on cytospin preparation of serous effusions: A cost-effective measure in resource-limited settings. Cytojournal 8: 14. 21829416 10.4103/1742-6413.83233 PMC3151400

[44] Fritschy JM (2008) Is my antibody-staining specific? How to deal with pitfalls of immunohistochemistry. Eur J Neurosci 28: 2365–2370. 19087167 10.1111/j.1460-9568.2008.06552.x

[45] Gown AM (2008) Current issues in ER and HER2 testing by IHC in breast cancer. Mod Pathol 21 Suppl 2S8–S15. 18437174 10.1038/modpathol.2008.34

