# Supplementary figures and images for: The Use of Sensitive Chemical Antibodies for Diagnosis: Detection of Low Levels of Epcam in Breast Cancer
Source: PLoS One. 2013 Feb 27;8(2):e57613. doi: 10.1371/journal.pone.0057613 (PMC3584034; doi:10.1371/journal.pone.0057613)

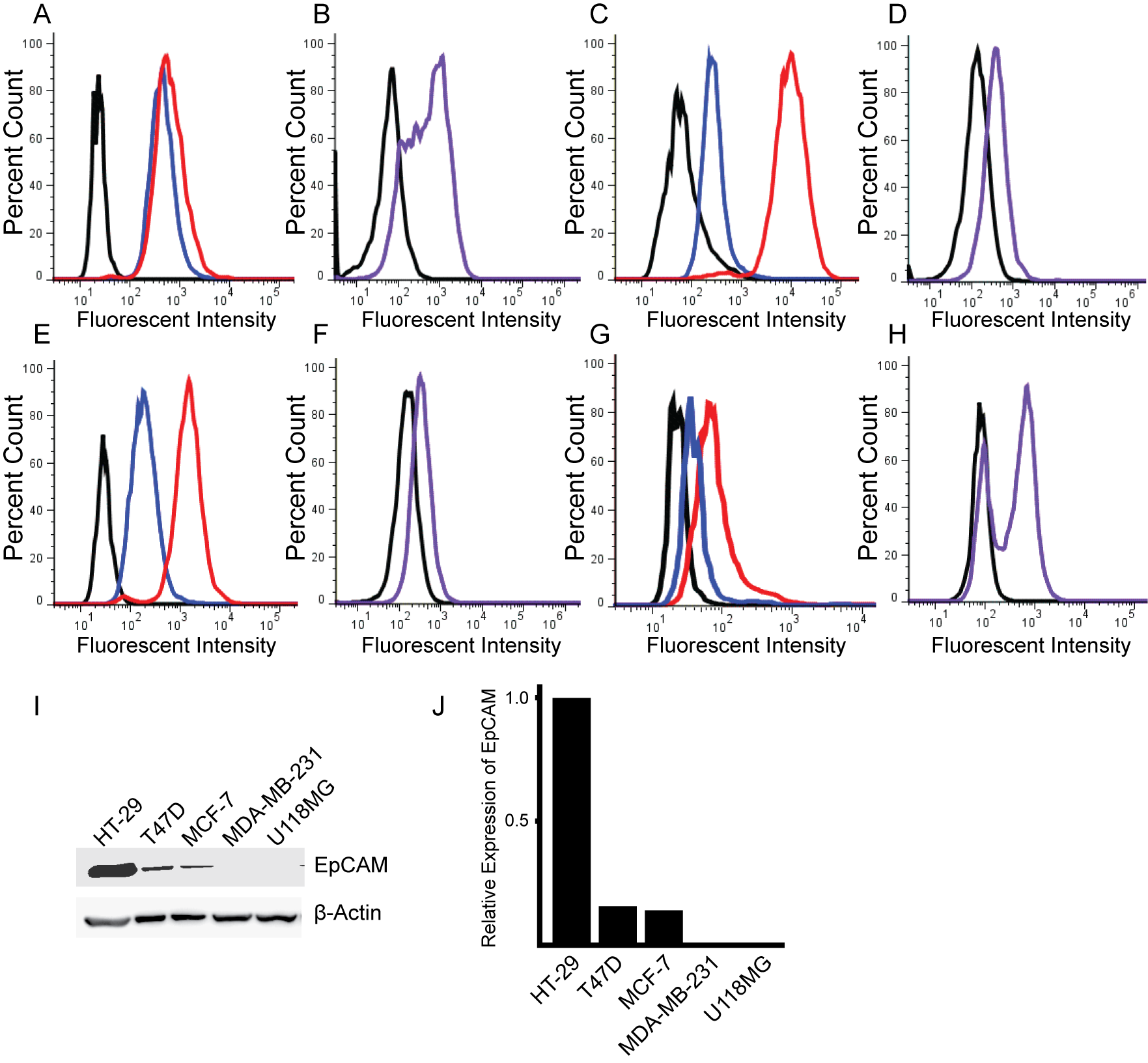

Supplement: Figure S1 — Determination of EpCAM expression by flow cytometric analysis and Western analysis. Flow cytometry was used to confirm EpCAM expression by EpCAM-FITC antibody and DT3 and Ep23 aptamers. A: Flow cytometry analysis of EpCAM expression of T47D with DT3 and Ep23 aptamers; B Flow cytometry analysis of EpCAM expression of T47D with EpCAM-FITC antibody; C: Flow cytometry analysis of EpCAM expression of MCF-7 with DT3 and Ep23 aptamers; D: Flow cytometry analysis of EpCAM expression of MCF-7 with EpCAM-FITC antibody; E: Flow cytometry analysis of EpCAM expression of MDA-MB-231 with DT3 and Ep23 aptamers; F Flow cytometry analysis of EpCAM expression of MDA-MB-231 with EpCAM-FITC antibody; G: Flow cytometry analysis of EpCAM expression of HT-29 with DT3 and Ep23 aptamers; H Flow cytometry analysis of EpCAM expression of HT-29 with EpCAM-FITC antibody. Black: negative control; Blue: DT3 aptamer (A, C, E, G); Red: Ep23 aptamer (A, C, E, G); Purple: EpCAM antibody (B, D, F, H). I: EpCAM expression was confirmed by Western analysis using the 323/A3 antibody; J: Relative expression of EpCAM was compared to β-actin. (TIF) [file pone.0057613.s001.tif]

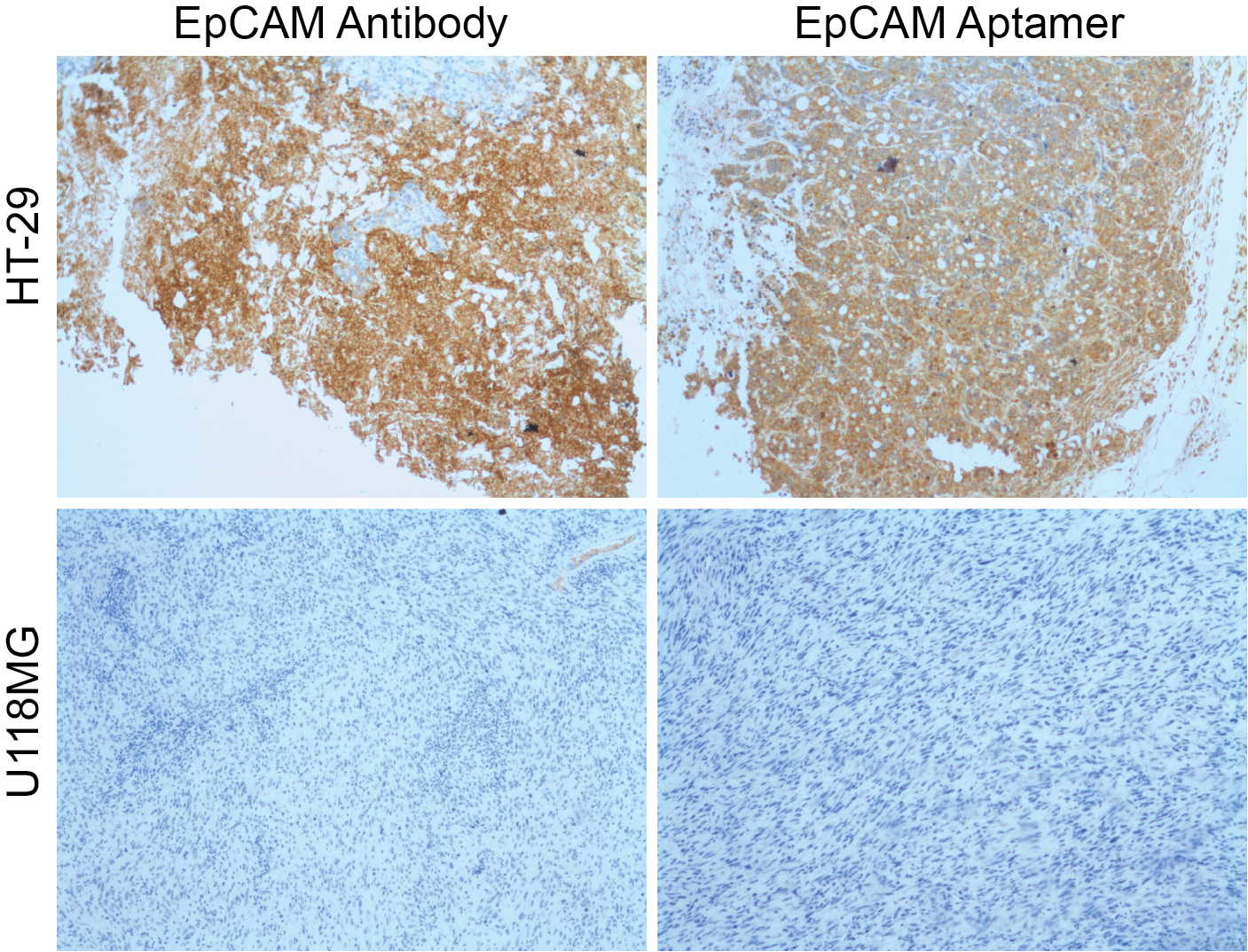

Supplement: Figure S2 — Representative images of positive and negative control slides for chromogenic staining. HT-29 and U118MG tissue sections were stained with either EpCAM antibody or Ep23 aptamer as part of each staining experiment of clinical breast cancer cases to confirm specificity of each staining reaction. (TIF) [file pone.0057613.s002.tif]
